# Genetic characterization of low-pathogenic avian influenza subtypes H10N6 and H10N7 from free-grazing ducks in Thailand

**DOI:** 10.14202/vetworld.2024.2166-2176

**Published:** 2024-09-28

**Authors:** Supanat Boonyapisitsopa, Supassama Chaiyawong, Kamonpan Charoenkul, Kitikhun Udom, Ekkapat Chamsai, Waleemas Jairak, Wikanda Tunterak, Napawan Bunpapong, Alongkorn Amonsin

**Affiliations:** 1 Department of Veterinary Public Health, Faculty of Veterinary Science, Chulalongkorn University, Bangkok, 10330, Thailand; 2 Emerging and Re-emerging Infectious Diseases in Animals, Center of Excellence, and One Health Research Cluster, Faculty of Veterinary Science, Chulalongkorn University, Bangkok, 10330, Thailand; 3 Veterinary Diagnostic Laboratory, Faculty of Veterinary Science, Chulalongkorn University, Bangkok, 10330, Thailand

**Keywords:** free-grazing ducks, genetic characterization, H10N6, H10N7, influenza A virus

## Abstract

**Background and Aim:**

Free-grazing duck (FGD) raising is a unique domestic duck production system that is widely practiced in several Asian countries, including Thailand. FGD is a significant reservoir for influenza A viruses (IAVs). In this study, we genetically characterized IAV-H10N6 and IAV-H10N7 isolated from avian influenza surveillance in FGDs in Thailand.

**Materials and Methods:**

We collected 640 swab samples from 29 FGD flocks located in 6 provinces of Thailand. IAVs were isolated from swab samples using egg inoculation. Hemagglutination test-positive samples were then subjected to IAV detection. Viral RNA was subjected to IAV detection using real-time reverse-transcription polymerase chain reaction (rRT-PCR) specific to matrix (M) gene. IAV subtypes were identified using the RT-PCR assay specific to all hemagglutinin and neuraminidase subtypes. Whole-genome sequencing of IAVs was performed to genetically characterize IAV-H10N6 and IAV-H10N7.

**Results:**

Our results showed that 41 (6.41%) samples tested positive for IAV using rRT-PCR specific to the M gene. Among these, only two IAVs were subtypes as IAV-H10N6 and IAV-H10N7 and were subjected to whole-genome sequencing. IAV-H10N6 and IAV-H10N7 belonged to the Eurasian lineage and did not show any evidence of reassortment from the North American lineage. The viruses exhibited low-pathogenic characteristics and preferred binding to avian-type receptors. Genetic analysis revealed no mutations in PB2 and M genes, unlike human IAV-H10N3 and IAV-H10N8, which exhibited increased virulence in mammals.

**Conclusion:**

IAV-H10N6 and IAV-H10N7 viruses have less potential as zoonotic viruses. However, IAV in FGDs should be monitored for novel reassortant or zoonotic viruses. This study provides information on the genetic characteristics and diversity of IAV-H10N6 and IAV-H10N7 that are circulated in FGDs in Thailand.

## Introduction

Influenza A virus (IAV) significantly threatens animal and human health. IAVs are classified into subtypes based on their surface glycoproteins, hemagglutinin (HA), and neuraminidase (NA). 18 HA subtypes (H1–H18) and 11 NA subtypes (N1–N11) have been identified. Waterfowl serve as natural reservoirs for IAVs and can transmit viruses to other avian and mammal species. IAVs can be classified as highly pathogenic avian influenza (HPAI) and low-pathogenic avian influenza (LPAI). HPAI viruses, such as HPAI-H5 and HPAI-H7, can lead to severe and sometimes fatal disease in birds, mammals, and humans. Conversely, LPAI viruses cause mild to asymptomatic infections in birds and mammals. However, specific LPAI subtypes, such as H6N1, H7N9, H10N3, and H10N8, are known to sporadically infect humans, raising concerns about their potential to cause zoonotic and pandemic outbreaks [1–3].

IAV subtype H10 has been reported in various species of wild birds and waterfowl worldwide. IAV-H10 can be classified into two lineages: the Eurasian and North American lineages. For example, multiple subtypes of IAV-H10 (H10N1, H10N6, H10N7, and H10N9) were isolated from poultry in live bird markets in Bangladesh in 2009 [4]. IAV-H10N3 was found in teals at a live bird market in Egypt in 2015 [5]. IAV-H10N7 was isolated from a poultry farm in Australia in 2010 and clustered with IAVs of North American lineage [6]. IAV-H10N4 was detected in wild birds (common teal and mallard ducks) in China in 2018 [7]. IAV subtypes H10N3, H10N7, and H10N8 were isolated from domestic ducks in live poultry markets in China during 2009–2010 [8]. IAV-H10N4 and IAV-H10N7 were isolated from wild birds in South Korea between 2019 and 2020 [9]. Moreover, it is known that IAV-H10 is occasionally infected in mammalian species. IAV-H10N4 infection was first reported in Sweden in 1984 in mink farms during a respiratory disease outbreak [10]. IAV-H10N7 infection caused influenza outbreaks and death in harbor seals in Denmark and Sweden in 2014 [11]. The first report of IAV-H10N7 infection in infants in Egypt was published in 2004 [12]. IAV-H10 was also detected in workers in poultry slaughterhouses in Australia in 2010, indicating the potential for human infection from slaughtering IAV-infected poultry [6]. In China, the novel H10N8 was isolated from a patient with severe pneumonia in 2013. The novel H10N8 strain was reassorted from multiple avian influenza viruses [1]. In China, in 2021–2022, IAV-H10N3 infections in humans were reported [3]. In China, in 2024, IAV-H10N5 infection was first reported in a patient with severe pneumonia [13].

In Thailand, poultry in live bird markets and free-grazing ducks (FGDs) are important reservoirs of IAVs. The previous studies by Chaiyawong *et al*. [14] and Jairak *et al*. [15] reported multiple IAV subtypes isolated from FGDs and poultry in live bird markets in Thailand. The novel IAV-H10N3 was isolated from ducks in a live bird market in 2010 [16]. This study aimed to isolate IAV-H10N6 and IAV-H10N7 from retrospective samples from avian influenza surveillance programs conducted in FGDs in Thailand. We conducted genetic characterization of Thai IAV-H10 to assess its genetic diversity.

## Materials and Methods

### Ethical approval and informed consent

This study was conducted under the approval of the Institute for Animal Care and Use Protocol (IACUC no. 13310025 and 1831105). Verbal consent was obtained from all FGD flock owners for sample collection. This study was conducted in compliance with the ARRIVE guidelines.

### Study period and location

This study was conducted from January 2014 to December 2015. FGD flocks located in 6 provinces of Thailand (Phitsanulok, Phichit, Sukhothai, Kamphaeng Phet, Ayutthaya, and Suphan Buri) were included in this study.

### Sample collection and virus isolation

**Figure-1:**
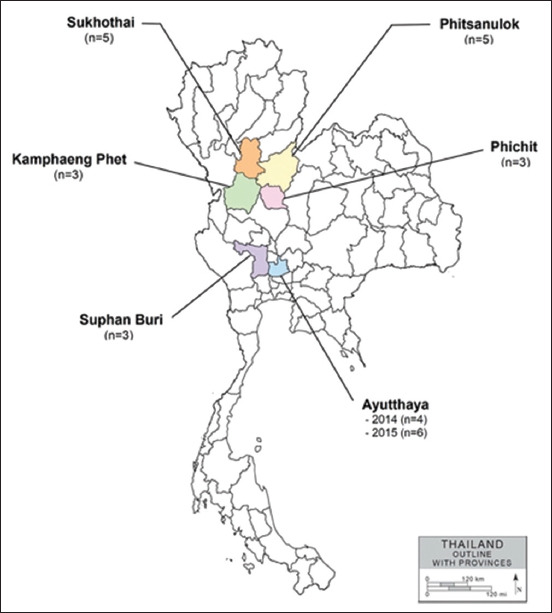
Map of free-grazing duck flocks by Province [Source: ^©^Copyright 2007 by World Trade Press.]

Oropharyngeal (OP) swabs and cloacal swabs (CS) were collected for avian influenza surveillance in FGDs in Thailand. FGD flocks (n = 29) located in six provinces in the central and lower northern regions of Thailand were selected based on the high density of FGD production and owner cooperation (Table-1 and Figure-1). IAVs were isolated from swab samples using egg inoculation, and the harvested allantoic fluid was subjected to hemagglutination test (HAT) [17]. HAT-positive samples were subjected to IAV detection using real-time reverse-transcription polymerase chain reaction (rRT-PCR).

**Table-1:** Description of sample collection and IAV identification in free-grazing ducks from 2014 to 2015.

Province	Region	Month and year	No. of FGD flocks	No. of FGDs	No. of swabs	HAT positive (%)	IAV positive (%)	IAV subtype
Phitsanulok	Northern	January-14	5	50	100	31 (31)	5 (5)	H10N7 (n = 1)
Sukhothai	Northern	February-14	5	50	100	17 (17)	2 (2)	-
Phichit	Northern	January-15	3	30	60	9 (15)	0	
Kamphaeng Phet	Northern	February-15	3	30	60	49 (81.67)	3 (5)	
Suphan Buri	Central	January-15	3	30	60	7 (11.67)	1 (1.67)	H10N6 (n = 1)
Ayutthaya	Central	December-14	4	40	80	17 (21.25)	0	-
Ayutthaya	Central	January-15	3	30	60	32 (53.33)	0	
Ayutthaya	Central	December-15	3	60	120	31 (25.83)	30 (25)	
			29	320	640	193 (30.16)	41 (6.41)	

IAV=Influenza A virus, FGD=Free-grazing duck, HAT=Hemagglutination test

### IAV detection and subtype identification

Viral RNA extraction from positive samples was performed using a commercial kit (Nucleospin® RNA virus, Macherey-Nagel, Germany) following the manufacturer’s protocol. Viral RNA was subjected to IAV detection using rRT-PCR specific to matrix (M) gene. The result was interpreted based on cycle threshold (Ct) values. Samples with a Ct value of <36 were considered positive, 36–40 were considered suspect, and >40 were considered negative. IAV subtypes were identified using RT-PCR specific to all HA (1–16) and NA (1–9) subtypes as described by Tsukamoto *et al*. [18]. First, the RNA of IAV-positive samples was converted to complementary DNA (cDNA) using the Improm-II™ Reverse Transcription System (Promega, Madison, WI, USA). IAV subtypes were identified using polymerase chain reaction (PCR) with primers specific to all HA (H1-H16) and NA (N1-N9) subtypes. In brief, the PCR mixture of 30 μL contained 1 μL of cDNA, 1× master mix buffer (TopTaq™, Qiagen, Hilden, Germany), 0.8 μM of primers for each subtype, and distilled water. The PCR process consisted of 94°C for 3 min and 35 cycles of 94°C for 30 s, 50°C (for H1-H15) or 45°C (for N1-N9) for 30 s, and 72°C for 30 s. Gel electrophoresis was performed using a 1.2% agarose gel with Red Safe in 0.5× tris-borate-EDTA buffer.

### IAV characterization and genetic analysis

Whole-genome sequencing of IAVs was carried out for genetic characterization of IAV-H10N6 and IAV-H10N7 using Sanger sequencing with the BigDye™ terminator v3.1 cycle sequencing kit (1^st^ Base Laboratories, Malaysia) and Oxford Nanopore sequencing with the Rapid sequencing kit (SQK-RAD004; ONT, UK). All eight segments of IAVs were amplified using PCR with TopTaq master mix (Qiagen) with specific primer sets and newly designed primers for IAV-H10N6 and IAV-H10N7 sequencing (Supplementary Table-1). Amplicons were purified using a Nucleospin® PCR clean-up kit. The purified PCR products were sequenced for Sanger sequencing using a BigDye™ Terminator v3.1 cycle sequencing kit (1^st^ Base Laboratories). SeqMan software v.5.03 (DNASTAR Inc., Madison, WI, USA) was used to validate and assemble the nucleotide sequences. For Oxford Nanopore sequencing, Minion flow cells with a Rapid sequencing kit (SQK-RAD004, ONT) were used. The DNA library and priming mix were processed using MinKNOW software (ONT) according to the manufacturer’s instructions. MinKNOW software was used for sequence reading and base-calling processes (minimum Qscore 7) to convert the Fast5 file to a Fastq file. The Fastq file was then mapped to the reference sequences using CLC Genomic Workbench 20.0 (Qiagen).

**Supplementary Table-1:** The designed primers for HA and NA genes sequencing.

Gene	Primer	Size	Temperature (°C)	Primer sequence (5’- 3’)
H10	H10-A1F	20	55.4	GTA ATA ATC GCG CTC CTT GG
	H10-A1R	20	57.7	TCT GCC TCA GTG CTT CTT CA
	H10-A2F	20	56.5	CAC CAG CTT GTG ATC TGC AT
	H10-A2R	20	57.7	CCC ACT GTT CTT GGT GAC AA
	H10-A3F	20	57.4	CCC AGG TCA ATG GAC AAA GT
	H10-A3R	20	55.4	GCC ATA TCG ATT GTG TGC TG
	H10-A4F	20	57.4	TCA GAC ACC AAA ACG CTG AG
	H10-A4R	20	54.9	CAG ATT GTC GAT CGC ATG TT
N6	N6-A1F	20	55.3	GCA AAA GCA GGG TGA AAA TG
	N6-A1R	20	57.3	AGC TCG GAA TGG GCT TCT AT
	N6-A2F	20	57.3	CTG CAG GAT GTT TGC TCT GA
	N6-A2R	20	59.4	CGG TTA GGA CCT TTG AGC AC
	N6-A3F	20	55.3	TCC TGA AAT GAT GAC CCA CA
	N6-A3R	24	54.2	AGA AAC AAG GGT GTT TTT CTT AAA
N7	N7-A61F-7	21	55.9	AGC AGG GTG ATT GAG AAT GAA
	N7-A61R-584	19	54.5	CAA TCC CAT CAT GGC AAG T
	N7-A62F-395	20	52.4	CCA ACA GGA TGC AAR ATG TA
	N7-A62R-1117	20	59.3	GGT CCT ACC AAG CCA TGT GT
	N7-A63F-912	24	54.7	GAG CAA AYA GGC CTA TTA TAG AAA
	N7-A63R-1460	21	54.0	GAA ACA AGG GTG TTT TTG CAT

The Basic Local Alignment Search Tool (BLAST) (NCBI, MD, USA) was used to compare the nucleotide identities of Thai IAV-H10. The nucleotide and amino acid sequences of each gene segment of Thai IAVs-H10 were compared with those of IAVs-H10 available in the GenBank. The nucleotide and deduced amino acid sequences of Thai IAVs-H10 were aligned with those of the reference IAVs of different subtypes from Eurasian and North American lineages using Muscle v.3.6 and MegAlign v.5.03 (DNASTAR Inc.) programs. Phylogenetic trees of the HA and NA genes of Thai IAV-H10N6 and IAV-H10N7 were generated using a neighbor-joining algorithm with 1000 bootstrap replications and the MEGA 11.0 program (https://www.megasoftware.net/ dload_win_beta). The references IAV-H10Nx, HxN6, and HxN7 available in the database were selected based on various geographic locations for phylogenetic analysis.

## Results

### IAV subtypes H10N6 and H10N7 isolated from FGDs in Thailand

FGD flocks (n = 29) located in six provinces of Thailand (Phitsanulok, Phichit, Sukhothai, Kamphaeng Phet, Ayutthaya, and Suphan Buri) were selected for sample collection during 2014–2015 (Table-1 and Figure-1). A total of 640 swab samples were collected from 320 FGDs, which included OP swabs (n = 320) and CS (n = 320). Our results showed that out of 640 samples tested using rRT-PCR (M gene), 41 (6.41%) were positive for IAV detection (Table-1). Only two of the 41 IAVs were subtyped as IAV-H10N7 and IAV-H10N6. The remaining 38 isolates could not be classified due to low viral titers (high Ct-value). IAV-H10N7 (CU-14284C) was isolated from cloacal FGD swabs from Phitsanulok in 2014, and IAV-H10N6 (CU-15857T) was isolated from OP FGD swabs from Suphan Buri in 2015. Both IAV-H10N7 and IAV-H10N6 were subjected to whole-genome sequencing. The nucleotide sequences of IAV-H10N6 and IAV-H10N7 were submitted to GenBank under accession numbers #PP683372-79 and #PP683380-87, respectively (Table-2).

**Table-2:** Description of Thai IAV-H10 from free-grazing ducks.

Virus	Strain name	Subtype	Date	Location	Source	GenBank#
CU-14284C (H10N7)	A/duck/Thailand/CU-14284C/2014	H10N7	January 2014	Phitsanulok	CS	PP683372-79
CU-15857T (H10N6)	A/duck/Thailand/CU-15857T/2015	H10N6	January 2015	Suphan Buri	OP	PP683380-87

IAV=Influenza A virus, OP=Oropharyngeal swab, CS=Cloacal swab

### Whole-genome sequences and genetic characteristics of IAV-H10N6 and IAV-H10N7

**Figure-2:**
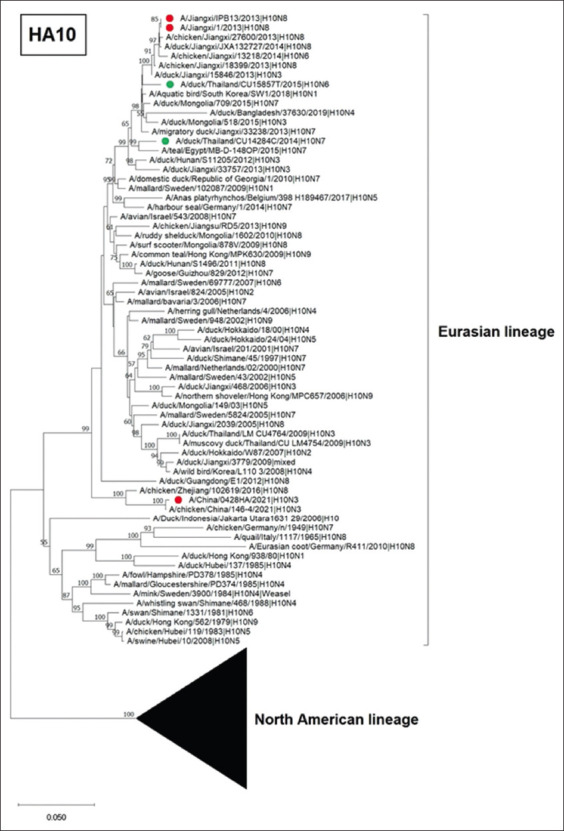
Phylogenetic tree of H10 gene of Thai IAV-H10. The phylogenetic tree was generated using the neighbor-joining algorithm with 1000 bootstrap replications in MEGA11.0. The green circles represent Thai IAV-H10N6 and IAV-H10N7, and the red circles represent IAVs isolated from humans. IAV=Influenza A virus.

For the HA gene, phylogenetic analysis showed that Thai IAV-H10N6 and IAV-H10N7 were clustered with the IAVs of the Eurasian lineage of LPAI (Figure-2). BLAST analysis showed that IAV-H10N6 was closely related to IAV-H10N1 (A/Aquatic bird/South Korea/SW1/2018), sharing 98.52% nucleotide identities. IAV-H10N7 was closely related to IAV-H10N7 (A/teal/Egypt/MB-D-148OP/2015) with 97.63% nucleotide identity (Supplementary Table-2). When comparing the H10 gene of Thai IAVs-H10 with those of human IAV-H10N8 and H10N3, which caused human infection in China in 2013 and 2021, IAV-H10N6 had 96.36% and 90.35% nucleotide similarities with human-H10N8 and human-H10N3, respectively. In addition, IAV-H10N7 had nucleotide similarities of 97.58% and 90.42% with human H10N8 and human H10N3, respectively. Furthermore, the H10 genes of IAV-H10N6 and IAV-H10N7 in this study were clustered separately from the IAV-H10N3 (CU-LM4754 and CU-4764) that was previously reported in Thailand in 2011 (91.13% and 91.32% nucleotide similarity), suggesting the genetic diversity of IAV-H10 circulating in Thailand (Table-3 and Figure-2).

**Supplementary Table-2:** BLAST results for the nucleotide identities of IAV-H10N7 and IAV-H10N6 in this study.

Gene	Virus having the highest nucleotide identity	Length (bp)	% Nucleotide identities
A/duck/Thailand/CU-14284C/2014(H10N7)			
PB2	A/mallard/Tumuji/TMJ-748/2013(H6N2)	2280	98.86
PB1	A/Anseriformes/Anhui/L25/2014(H1N1)	2283	99.87
PA	A/Anseriformes/Anhui/L167/2014(H1N1)	2143	98.93
HA10	A/teal/Egypt/MB-D-148OP/2015(H10N7)	1686	97.63
NP	A/duck/Anhui/FA258/2013(H1N3)	1454	99.52
NA7	A/mallard duck/Georgia/1/2014(H10N7)	1377	97.89
M	A/common teal/Nanji/NJ-280/2013(H6N1)	953	99.79
NS	A/common teal/Nanji/NJ-280/2013(H6N1)	838	99.40
A/duck/Thailand/CU-15857T/2015(H10N6)			
PB2	A/common teal/Nanji/NJ-280/2013(H6N1)	2280	98.86
PB1	A/duck/Vietnam/LBM798/2014(H3N6)	2274	99.25
PA	A/duck/Jiangxi/5461/2014(H7N3)	2151	99.30
HA10	A/Aquatic bird/South Korea/SW1/2018(H10N1)	1686	98.52
NP	A/duck/Tottori/K191/2015(H5N3)	1497	98.93
NA6	A/duck/Thailand/CU-11825C/2011(H3N6)	1434	97.77
M	A/chicken/Wuhan/WHJF/2014(H5N2)	982	99.59
NS	A/common teal/Nanji/NJ-280/2013(H6N1)	818	99.88

IAV=Influenza A virus

**Table-3:** Nucleotide similarities between Thai IAV-H10N7 and IAV-H10N6 and the reference IAV-H10.

Virus	Host	Location	Year	% Nucleotide similarity							

				PB2 (2189 bp)	PB1 (2225 bp)	PA (2109 bp)	HA (1644 bp)	NP (1454 bp)	NA (1377 bp)	M (953 bp)	NS (817 bp)
A/duck/Thailand/CU-14284C/2014 (H10N7)	Duck	Thailand	2014	100	100	100	100	100	100	100	100
This study											
A/duck/Thailand/CU-15857T/2015 (H10N6)	Duck	Thailand	2015	91.48	96.53	95.72	96.23	98.75	N/A	94.87	99.26
Closely related viruses											
A/teal/Egypt/MB-D-148OP/2015 (H10N7)	Teal	Egypt	2015	91.92	98.59	97.48	97.52	94.98	98.65	98.94	96.73
A/mallard duck/Georgia/1/2014 (H10N7)	Duck	Georgia	2014	92.68	96.82	95.25	95.72	97.76	98.82	99.16	97.50
A/aquatic bird/South Korea/SW1/2018 (H10N1)	Bird	South Korea	2018	93.15	95.13	94.53	97.33	98.89	N/A	97.65	97.12
A/duck/Thailand/CU-11825C/2011(H3N6)	Duck	Thailand	2011	N/A	N/A	N/A	N/A	N/A	N/A	N/A	N/A
Reference viruses											
A/Jiangxi/IPB13/2013 (H10N8)	Human	China	2013	89.87	88.76	88.52	96.36	91.05	N/A	89.98	89.43
A/China/0428/2021 (H10N3)	Human	China	2021	88.17	87.39	87.50	90.35	90.39	N/A	89.21	87.43
A/swine/Hubei/10/2008 (H10N5)	Swine	China	2008	89.69	90.39	89.89	87.24	92.54	N/A	97.21	94.48
A/harbor seal Germany 1/2014 (H10N7)	Seal	Germany	2014	92.46	95.95	95.20	94.73	96.75	98.10	98.08	96.86
A harbor seal, Canada/OTH-52-1/2021 (H10N7)	Seal	Canada	2021	80.53	85.56	88.04	93.44	90.46	97.02	93.11	94.62
A/turkey/MN/3/1979(H10N7)	Turkey	USA	1979	82.15	87.06	87.22	77.85	88.32	82.94	93.70	92.16
A/muscovy duck/Thailand/CU-LM4754/2009(H10N3)	Duck	Thailand	2009	85.98	96.20	94.12	91.50	94.29	N/A	97.87	96.86
A/duck/Thailand/CU-15857T/2015 (H10N6)	Duck	Thailand	2015	100	100	100	100	100	100	100	100
A/duck/Thailand/CU-14284C/2014 (H10N7)	Duck	Thailand	2014	91.48	96.53	95.72	96.23	98.75	N/A	94.87	99.26
Closely related viruses											
A/teal/Egypt/MB-D-148OP/2015 (H10N7)	Teal	Egypt	2015	95.35	95.52	96.23	96.30	94.83	N/A	95.20	97.37
A/mallard duck/Georgia/1/2014 (H10N7)	Duck	Georgia	2014	94.61	96.38	95.93	95.65	97.76	N/A	94.98	98.14
A/Aquatic bird/South Korea/SW1/2018 (H10N1)	Bird	South Korea	2018	92.90	95.37	95.52	98.46	98.68	N/A	96.00	97.63
A/duck/Thailand/CU-11825C/2011(H3N6)	Duck	Thailand	2011	N/A	N/A	N/A	N/A	N/A	97.70	N/A	N/A
Reference viruses											
A/Jiangxi/IPB13/2013 (H10N8)	Human	China	2013	90.19	89.30	88.82	97.58	91.06	N/A	90.71	89.88
A/China/0428/2021 (H10N3)	Human	China	2021	88.08	87.54	88.02	90.42	90.15	N/A	89.71	87.25
A/swine/Hubei/10/2008 (H10N5)	Swine	China	2008	89.54	91.22	90.68	86.44	92.24	N/A	95.88	95.03
A/harbor seal/Germany/1/2014 (H10N7)	Seal	Germany	2014	95.15	96.38	95.67	94.66	96.02	N/A	95.10	97.38
A/harbor seal/Canada/OTH-52-1/2021 (H10N7)	Seal	Canada	2021	80.86	85.83	88.57	94.26	90.31	N/A	92.49	95.29
A/turkey/MN/3/1979 (H10N7)	Turkey	USA	1979	81.03	88.38	87.86	78.20	87.91	N/A	93.67	92.45
A/muscovy duck, Thailand/CU-LM4754/2009 (H10N3)	Duck	Thailand	2009	86.19	97.57	94.96	91.31	94.52	N/A	95.55	97.12

IAV=Influenza A virus

**Figure-3:**
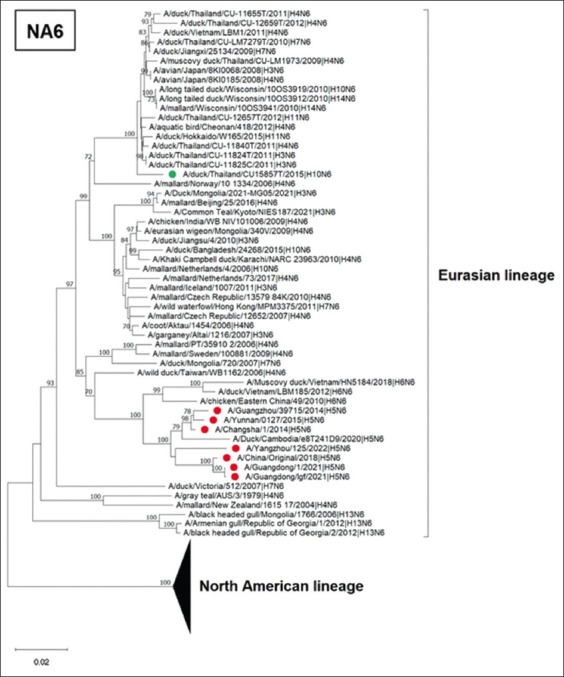
Phylogenetic tree of N6 gene of Thai IAV-H10N6. The phylogenetic tree was generated using the neighbor-joining algorithm with 1000 bootstrap replications in MEGA11.0. The green circles represent Thai IAV-H10N6 and IAV-H10N7, and the red circles represent IAVs isolated from humans. IAV=Influenza A virus.

**Figure-4:**
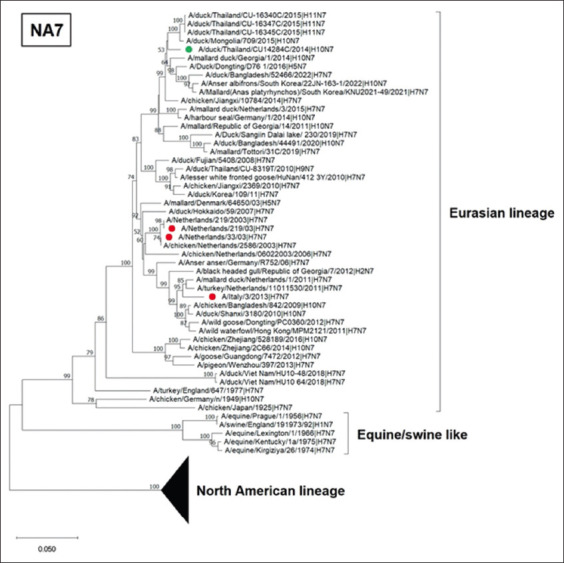
Phylogenetic tree of N7 gene of Thai IAV-H10N7. The phylogenetic tree was generated using the neighbor-joining algorithm with 1000 bootstrap replications in MEGA11.0. The green circles represent Thai IAV-H10N6 and IAV-H10N7, and the red circles represent IAVs isolated from humans. IAV=Influenza A virus.

For the NA gene, the phylogenetic tree of N6 contains two lineages: the North American and Eurasian lineages of LPAI. The Thai IAV-H10N6 was grouped in the Eurasian lineage. It was noted that IAV-H10N6 was clustered separately from IAV-H5N6, which has caused infection in humans in China since 2014 (Figure-3). The phylogenetic tree of the N7 gene shows that Thai IAV-H10N7 was also clustered in the Eurasian lineage. This virus was grouped with the N7 gene (H11N7) previously found in ducks in Thailand in 2015. However, our Thai IAV-H10N7 was separated from IAV-H7N7 previously reported in humans in Europe (Figure-4).

### Genetic analysis of IAV H10N6 and IAV H10N7

Genetic analysis of IAV-H10N6 and IAV-H10N7 was conducted by comparing the deduced amino acid sequences of the HA, NA, PB2, PA, NP, M, and NS genes. Our results showed that all Thai IAVs-H10 had an HA cleavage site of “PELMQGR/GLF” without any multiple basic amino acids, suggesting that Thai IAVs-H10 in this study possessed an LPAI characteristic. The receptor binding sites of both IAV-H10N6 and H10N7 contained Q226 and G228, indicating that these viruses preferred to bind with the avian receptor (α 2,3-linked sialic acid receptor). Both IAVs-H10 did not contain an amino acid substitution for the NA gene at positions 119, 222, and 292 that were linked to NA inhibitor resistance. At position 627 of the PB2 gene, Thai IAVs-H10 contained glutamic acid (E), similar to the other avian H10 viruses, whereas the human H10N8 virus contained glycine (G). For M gene genetic analysis, IAVs-H10 in this study contained L26, V27, A30, S31, and G34, similar to the other avian IAVs, whereas both human IAVs, H10N8 and H10N3, exhibited S31N substitution, which was associated with amantadine resistance (Table-4).

**Table-4:** Genetic analysis of Thai IAV-H10N6 and H10N7 viruses.

Virus	Subtype	Host	HA gene											NA gene		
	
			Cleavage site	Receptor binding site										Neuraminidase Inhibitor Resistance		
		
			320-329	Y95	W151	H183	E190	K191	L194	Q226L	S227	G228S	R229	E119V	I222L	R292K
Eurasian lineage consensus	H10Nx	Avian	PELMQGR	Y	W	H	E	K	L	Q	S	G	R	N/A	N/A	N/A
North American lineage consensus	H10Nx	Avian	PEVVQGR	Y	W	H	E	K	L	Q	S	G	R	N/A	N/A	N/A
A/Jiangxi/IPB13/2013	H10N8	Human	PELIQRG	Y	W	H	E	K	L	Q	S	G	R	N/A	N/A	N/A
A/China/0428/2021	H10N3	Human	PEIIQGR	Y	W	H	E	K	L	Q	S	S	I	E	I	R
A/Harbor Seal/Canada/OTH-52-1/2021	H10N7	Seal	PELIQGR	Y	W	H	E	K	L	Q	S	G	R	E	I	R
A/swine/Hubei/10/2008	H10N5	Swine	PEIIQGR	Y	W	H	E	K	L	Q	S	G	R	N/A	N/A	N/A
A/duck/Fujian/1761/2010	H10N3	Duck	PEIMQGR	Y	W	H	E	K	L	Q	S	G	R	E	I	R
A/duck/Bangladesh/24268/2015	H10N6	Duck	PELMQGR	Y	W	H	E	K	L	Q	S	G	R	E	1	R
A/duck/Thailand/CU-LM4754/2009	H10N3	Duck	PEIIQGR	Y	W	H	E	K	L	Q	S	G	R	E	I	R
This study																
CU-14284C	H10N7	Duck	PELMQGR	Y	W	H	E	K	L	Q	S	G	R	E	I	R
CU-12658T	H10N6	Duck	PELMQGR	Y	W	H	E	K	L	Q	S	G	R	E	I	R

**Virus**	**Subtype**	**Host**	**PB2 gene**			**PA gene**		**NP gene**		**M2 gene**					**NS1 gene**	
			
			**Virulence determinant**			**Increased virulence**		**Species adaptation**		**Amantadine resistance**					**Increased virulence**	
			
			**E627K**	**D701N**	**Q591K**	**353R**	**K356R**	**N319K**		**L26F**	**V27A**	**A30T**	**S31N**	**G34E**	**D92E**	**80-84 deletions**

Eurasian lineage consensus	H10Nx	Avian	E	D	Q	K	K	N		L	V	A	S	G	D	No deletion
North American lineage consensus	H10Nx	Avian	E	D	Q	K	K	N		L	V	A	S	G	D	No deletion
A/Jiangxi/IPB13/2013	H10N8	Human	K	D	Q	K	R	N		L	V	A	N	G	D	No deletion
A/China/0428/2021	H10N3	Human	E	D	K	K	R	N		L	V	A	N	G	D	No deletion
A/Harbor Seal/Canada/OTH-52-1/2021	H10N7	Seal	E	N	Q	K	K	K		L	V	A	S	G	D	No deletion
A/swine/Hubei/10/2008	H10N5	Swine	E	N	Q	K	K	N		L	V	A	S	G	D	No deletion
A/duck/Fujian/1761/2010	H10N3	Duck	E	D	Q	K	K	N		L	V	A	S	G	D	No deletion
A/duck/Bangladesh/24268/2015	H10N6	Duck	E	D	Q	K	K	N		L	V	A	S	G	D	No deletion
A/duck/Thailand/CU-LM4754/2009	H10N3	Duck	E	D	Q	K	K	N		L	V	A	S	G	D	No deletion
This study																
CU-14284C	H10N7	Duck	E	D	Q	K	K	N		L	V	A	S	G	D	No deletion
CU-12658T	H10N6	Duck	E	D	Q	K	K	N		L	V	A	S	G	D	No deletion

IAV=Influenza A virus

## Discussion

FGD raising is a unique domestic duck production system commonly practiced in several Asian countries, including Thailand, Vietnam, Indonesia, and China. FGDs are broiler or layer domestic ducks that roam freely and scavenge on rice paddy fields. Farmers move their FGD flocks from one grazing field to another, covering long distances across districts, provinces, and sometimes even countries. FGD flocks are transported on foot or by vehicles such as trucks and boats. It has been observed that FGD transport vehicles often lack proper cleaning or disinfection, which increases the risk of pathogen transmission between flocks. FGDs are an important reservoir for IAVs. FGDs are frequently fed in natural wetlands or rice paddy fields where domestic and wild birds coexist, increasing the probability of animal interfaces and IAV transmission. Young FGDs are susceptible to IAVs, which increase the risk of IAV transmission [19].

Several subtypes of LPAI viruses have been identified in FGDs. For instance, in Bangladesh, in 2015–2016, LPAI-H3N6, H7N1, H7N5, H7N9, and H15N9 were isolated from FGDs. Replication and pathogenic studies of H7N1 and H7N9 viruses in mallard ducks showed that these viruses replicated and shed with high titers, but no clinical signs were observed [20]. In Thailand, LPAI-H11N6, H11N7, and H11N9 viruses were isolated from FGDs. The viruses exhibited LPAI characteristics [14]. In Bangladesh, the novel reassortant LPAI-H9N9 was isolated from FGDs in 2020. Genetic analysis revealed that the HA and NA genes were closely related to IAVs isolated from South Korea, whereas other internal genes were closely related to IAVs isolated from Bangladesh [21].

LPAI-H10 viruses are known to infect not only avian species but also mammals, including humans. For example, during the LPAI-H10N7 outbreak at Australian poultry farms in 2010, slaughterhouse workers were found to have been infected with the virus. Infected humans had mild respiratory symptoms and conjunctivitis but showed no evidence of seroconversion [6]. In China, in 2013, a report of LPAI-H10N8 infection in a patient who had visited a live poultry market and suffered from fever, cough, pneumonia, and death was published. The LPAI-H10N8 exhibited all eight genes originating from avian viruses with a preference for HA binding to avian-like receptors. However, the substitution of Lysine at position PB2-627 suggests that the virus adapts to a mammalian host [1]. In China, in 2021, a patient was reported to have been infected with LPAI-H10N3, which caused fever and respiratory symptoms. Interestingly, the patient had not visited a live poultry market or interacted with poultry. Similarly, another case of LPAI-H10N3 infection in a patient was reported in China in June 2022 [3, 22]. In January 2024, LPAI-H10N5 coinfection with seasonal human H3N2 virus was detected in a patient in China**.** It has been noted that most human cases of IAV infection have a history of exposure to birds before illness onset, but there is no evidence of human-to-human transmission [13].

Several LPAI-H10 subtypes are also found in wild birds and domestic poultry. However, there are no reports of IAV-H10 viruses circulating in FGDs. For example, in eastern China in 2020, migratory birds on the East Asia-Australasia migratory flyway were found to carry LPAI-H10N4 and H10N8 viruses. The viruses exhibited an HA gene closely related to the North American lineage, whereas the NA and internal genes originated from migratory birds in Eurasia, suggesting evidence of reassortant IAVs in migratory birds [23]. Some IAV-H10 from birds have shown potential for replication in mammals. For example, IAVs-H10N1, H10N6, H10N7, and H10N9 were isolated from poultry in live bird markets in Bangladesh and replicated in mice. Moreover, H10N1, H10N6, and H10N7 IAVs cause mortality in mice [20]. IAV-H10N1 from aquatic birds in South Korea can be experimentally infected in mice and ferret models [24]. Some studies reported IAV-H10 infection in mammals. IAVs-H10N7 caused influenza and mortality in harbor seals in Denmark and Sweden in 2014 [11]. Recently, reassortant IAV-H10N7 was detected in harbor seal in Canada. The virus contained the M, PA, PB1, and PB2 genes of the North American lineage and the HA, NA, NP, and NS genes of the Eurasian lineage [25].

In this study, phylogenetically, the HA and NA genes of H10N6 and H10N7 viruses were grouped in the Eurasian lineage. Similarly, internal genes were closely related to IAVs in the Eurasian lineage. There was no evidence of reassortment in IAV-H10 in this study. Unlike other IAV-H10 strains reported in the previous studies by Wang *et al*. [23] and Berhane *et al*. [25], some gene segments were reassorted from the North American lineage. Genetic analysis showed that the HA cleavage sites of Thai IAV-H10N6 and H10N7 did not contain multiple basic amino acids, indicating LPAI characteristics. Similarly, IAV-H10N3 was previously isolated from poultry in a live bird market in Thailand, and it was clustered in the Eurasian lineage [16].

Genetic analysis of Thai IAV-H10N6 and H10N7 revealed that they contained receptor-binding properties (Q226 and G228) similar to those of avian viruses and human IAV-H10N3 and H10N8 viruses. Moreover, human IAV-H10N3 and H10N8 viruses tended to bind to avian-type receptors rather than human-type receptors [26]. The Thai IAV-H10N7 and H10N6 viruses contained no mutations at positions NA-119, 222, and 292, indicating that these viruses are sensitive to Oseltamivir. Moreover, these viruses did not contain amino acid substitutions at positions 26, 27, 30, 31, and 34 of the M gene, similar to avian IAVs. On the other hand, human IAV-H10N3 and H10N8 viruses contain 31N, which indicates resistance to Amantadine. The PB2 genes of IAV-H10N6 and H10N7 viruses encode for E627 and D701. In contrast, H10N8 in humans contained PB2-627K, suggesting increased virulence in mammals and humans. In an experimental study, human IAV-H10N8 with PB2-627K was associated with greater virulence in mammals [27].

In Thailand, FGDs are significant reservoirs of IAVs. The previous study by Chaiyawong *et al*. [14] reported multiple subtypes of LPAI viruses in FGDs. FGDs share habitats and grazing areas with wild birds and domestic animals; thus, they can act as sources of IAV transmission. In addition, FGDs can cause the reassortment of IAVs. This is because FGDs frequently move and cover large distances, making it easier for IAVs to spread and transmit. Therefore, surveillance of LPAI viruses in FGDs is important for monitoring the genetic diversity of IAVs and potential reassortant viruses.

## Conclusion

This study provides information about the genetic characteristics of IAV-H10N6 and IAV-H10N7 that are circulated in FGDs in Thailand. These viruses belonged to the Eurasian lineage and showed no evidence of reassortment from the North American lineage. Both IAV-H10N6 and IAV-H10N7 prefer binding avian-type receptors and exhibit low-pathogenic characteristics. Genetic analysis revealed no mutations in PB2 and M genes, unlike in humans H10N3 and H10N8, which indicated potentially increased virulence in mammals. Therefore, these IAV-H10 viruses have less potential as zoonotic viruses. However, IAV-H10 should be monitored for potential novel zoonotic or reassortant viruses in the future.

## Data Availability

The authors declare that the data supporting the findings of this study are available on request from the first author. The nucleotide sequence data that support the findings of this study are openly available in the GenBank database at https://www.ncbi.nlm.nih.gov/genbank/, under accession numbers #PP683372-79, and PP683380-87.

## Authors’ Contributions

SB, SC, WJ, WT, KU, EC, and NB: Virus isolation, molecular detection, whole genome characterization, and phylogenetic analysis. SC, KC, KU, and EC: Genome sequencing and phylogenetic analysis. SB: Drafted the manuscript. AA: Designed the study, analyzed data, and revised the manuscript. All authors have read, reviewed, and approved the manuscript.
